# Mental health treatment use and perceived treatment need among suicide planners and attempters in the United States: between and within group differences

**DOI:** 10.1186/s13104-015-1269-7

**Published:** 2015-07-16

**Authors:** Namkee G Choi, Diana M DiNitto, C Nathan Marti

**Affiliations:** University of Texas at Austin School of Social Work, 1925 San Jacinto Blvd, D3500, Austin, TX 78712 USA

**Keywords:** Suicidal thoughts, plans, and attempts, Major depressive disorder, Mental health treatment

## Abstract

**Background:**

Despite many previous studies of suicidal ideation and/or attempts, little research has examined mental health treatment use and perceived treatment need among and within groups of ideators and/or attemptors. We examined mental health treatment use and perceived treatment need in four groups of US adults who had serious suicidal ideation: (1) no suicide plan/no attempt; (2) planned/no attempt; (3) no plan/attempted; and (4) planned/attempted.

**Methods:**

We compared ideators and nonideators using the 154,923 U.S. residents aged 21 and older who participated in the 2008–2012 National Survey on Drug Use and Health (NSDUH). We then employed logistic regression analyses to discern factors associated with treatment use and perceived treatment need among and within the four groups of ideators (N = 7,348).

**Results:**

More than 30% of ideators who made suicide plans and/or attempted suicide received no treatment before or after planning or attempting. Racial/ethnic minorities had lower odds of treatment use in all four groups, but major depression significantly increased the odds in all but the no plan/attempted group. Treatment use and substance use disorder increased the odds of perceived need in all four groups.

**Conclusions:**

The four groups have different rates of treatment access and perceived treatment need that do not appear to be commensurate with their risk level. The findings underscore the importance of treatment access for all those at-risk of suicide, especially racial/ethnic minorities and those of lower SES.

## Background

Suicide is a serious public health problem globally and in the United States [1, 2]. Between 2007 and 2008, an estimated 8.3 million (3.7%) US adults (aged 18+ years) had suicidal thoughts, 2.2 million (1%) made suicide plans, and one million (0.5%) made a (nonfatal) suicide attempt [3]. There is one completed suicide for every 25 attempted suicides [4]. In 2010, suicide resulted in more than 38,000 deaths in the US [2]. Suicide was a top four cause of death among age groups between 15 and 54 years and the eighth leading cause among the 55–64 age group [2]. Although suicide is not a top ten cause of death among the 65+ age group (most deaths in this age group are due to medical conditions), men aged 65+ have one of the highest suicide rates in the US [5].

The US prevalence of suicidal ideation, plans, and attempts has remained relatively constant over the past two decades, and psychiatric disorder prevalence among those at risk of suicide is high. The 1990–1992 National Comorbidity Survey (NCS) and the 2001–2003 National Comorbidity Survey Replication (NCS-R) show that among those aged 18–54, more than 80% of suicide ideators, planners, and attempters met criteria for one or more mental disorders as described in the *Diagnostic and Statistical Manual of Mental Disorders (DSM*-*III*-*R* or -*IV)* in the previous 12 months [6]. Major depression was the most common individual disorder among ideators (42% in the NCS and 39% in the NCS-R), while anxiety disorders were the most common class of disorders (63% in the NCS and 61% in the NCS-R). Substance use disorders were also common (30% in the NCS and 19% in the NCS-R). Another study, based on the 2000–2001 Healthcare for Communities Survey of 7,896 respondents aged 25 and older found that of the 3.6% with suicidal ideation, 74% had a probable psychiatric disorder [7].

Individuals with suicidal thoughts and behaviors are more likely to use mental health treatment and perceive a need for it than those without; however, many do not access treatment and do not perceive a need for it [6–11]. For example, in the NCS-R, 44% of ideators-nonattempters and 61% of attempters used some type of specialty mental health treatment (i.e., treatment by a psychiatrist or a nonphysician mental health specialist such as a psychologist or social worker in a mental health specialty setting) [6]. More recent (2008–2012) US National Surveys on Drug Use and Health (NSDUH) found that about 42% of ideators and 56% of attempters aged 18 and older received mental health treatment; however, more than half of treatment users perceived unmet treatment need, while only one-fifth to one-fourth of those who did not receive treatment perceived treatment need [12, 13].

Interviews with 55,302 individuals aged 18 and older representing 21 nationally representative samples from the World Health Organization’s World Mental Health Surveys indicate that low perceived need was the most important reason for not seeking help (58%), followed by attitudinal barriers such as the wish to handle the problem alone (40%) and structural barriers such as financial concerns (15%) [2]. Although a higher proportion of attempters than nonattempters access mental health treatment, another US epidemiologic study found that attempters were more likely to be hospitalized than nonattempters but not more likely to use other forms of treatment [14]. Previous studies also found that ideators or attempters who were male, younger, of a race or ethnicity other than non-Hispanic white, uninsured, and who did not have a depression diagnosis were less likely to seek mental health treatment [12, 15].

Given ideators’ need for mental health treatment and low treatment use rates, more research is needed to better understand factors associated with treatment use and perceived treatment need among those who may have different levels of suicide risk. Focusing on US adults aged 21 and older, the present study first compared ideators with nonideators (i.e., those who did not report serious suicidal ideation). Second, the study compared four groups of ideators ( [1] those who neither planned nor attempted suicide; [2] those who planned but did not attempt suicide; [3] those who did not plan but attempted suicide; and [4] those who made plans and attempted suicide) with respect to their sociodemographic and clinical characteristics. Third, it examined whether the four ideator groups significantly differed in treatment use and perceived need, controlling for predisposing, enabling, and other need factors (between-group differences). Fourth, it examined predisposing, enabling, and need factors for treatment use within each of the four ideator groups (within-group differences).

The conceptual framework was Andersen’s behavioral model of health service use [16], which posits factors that impact health service access and utilization. *Predisposing factors* include individuals’ demographic characteristics and attitudes, values, and knowledge about health and health services that might impact their perceptions of need and health service use. For example, studies have found that racial/ethnic minority older adults and men, especially older men, are significantly less likely than non-Hispanic Whites to perceive a need for and use mental health care due to a higher level of stigma associated with mental health problems, cultural values and beliefs that emphasize the importance of self-sufficiency and religious coping, and more negative attitudes toward professional mental health treatment use [17–22]. *Enabling factors* include social support, income, insurance coverage, service availability and accessibility, transportation to treatment, and waiting times. A study based on the Baltimore Epidemiologic Catchment Area Cohort found that higher levels of social support from spouse/partner, relatives, and friends during a life event were generally associated with less specialty psychiatric service use [23]. Likewise, employed individuals may be less likely to use mental health services because they are more likely to be socially connected. College education and higher income were also positively associated with mental health treatment use [20]. As for *need factors*, previous studies show that mental illness and substance abuse/dependence diagnoses, severe symptom severity, and past mental health service use were strong predictors of perceived need and/or treatment use in both the general population and suicide ideators [12, 15, 24, 25]. In sum, although need factors have been found to be the most important predictors for treatment use, they are not sufficient conditions for treatment use. Predisposing and enabling factors, along with need factors, are significant determinants of treatment use.

The study hypotheses were: (H1) Compared to those who neither planned nor attempted suicide, the other three ideator groups will be more likely to (a) have used mental health treatment, and (b) perceive treatment need; and (H2) within each ideator group, those who are younger (aged 21–25) and older (65+ years), males, racial/ethnic minorities, married, have less than a college education, nonemployed, low income, and had an insurance coverage gap will have decreased odds of mental health treatment use; and those with major depressive episode (MDE) and substance use disorder (SUD) will have increased odds. This study adds to research on suicide by examining factors associated with mental health treatment use and perceived treatment need among four groups of suicide ideators.

## Methods

### Data and sample

Data came from the public use files of the 2008–2012 National Survey on Drug Use and Health (NSDUH). The annual NSDUH series measures substance use prevalence among the civilian, non-institutionalized, U.S. population aged 12+ years [26]. Questions about mental disorders, suicide, and mental health and substance abuse treatment are also asked of respondents aged 18+. Respondents were interviewed in private at their residence using audio computer-assisted self-interview, computer-assisted personal interview, and computer-assisted self-interview methods to increase the level of honest reporting of illicit drug use and other sensitive behaviors [26].

To increase the study’s power to detect low frequency events (e.g., suicide attempts), we combined 5 years of data. The survey sampling and data collection methods for the variables examined in this study were the same for all five survey years. A total of 283,049 respondents completed the survey from 2008 to 2012. The present study included all 154,923 respondents aged 21+ and focused on the 7,348 who self-reported having serious suicidal thoughts in the past year to examine study questions. NSDUH’s multi-stage area probability sampling design made any duplication of survey respondents unlikely in the 5 years of survey data.

### Ethics

The NSDUH’s data collection protocol was approved by the institutional review board at the Research Triangle Institute (RTI) International. RTI International is an independent, nonprofit research organization dedicated to conducting research that improves the human condition. NSDUH data files used for this study were downloaded from the US Substance Abuse and Mental Health Service Administration data archive website (http://www.icpsr.umich.edu/icpsrweb/SAMHDA/download).

### Measures

*Serious suicidal thoughts/ideation* were assessed with a question: “*At any time in the past 12* *months… including today, did you seriously think about trying to kill yourself?*” (yes = 1 [ideators] or no = 0 (nonideators]). The question did not elaborate on the meaning of “seriously”). For ideators, *suicide plans and attempts* were assessed with two questions, respectively; “*During the past 12* *months, did you make any plans to kill yourself?*”; “*During the past 12* *months, did you try to kill yourself?*” Responses to these questions (yes = 1 or no = 0) were used to classify the ideators into four groups: (1) no plan/no attempt; (2) planned/no attempt; (3) no plan/attempted; and (4) planned/attempted.

*Mental health treatment use* refers to any inpatient or outpatient treatment/counseling for “*any problem with emotions, nerves, or mental health (not including treatment for alcohol or drug use)*” during the past 12 months. Respondents were also asked if they received “treatment, counseling, or support” from acupuncturists or acupressurists, chiropractors, herbalists, in-person support group or self-help group, Internet support group or chat room, spiritual or religious advisors (e.g., a pastor, priest, rabbi), telephone hotlines, and massage therapists. In the present study, mental health treatment use refers to any treatment received from inpatient or outpatient settings and/or these alternative sources. Because respondents were asked about suicidal thoughts/behaviors and mental health treatment use in the past 12 months, the temporal order of these variables cannot be determined (i.e., treatment use may have preceded or followed suicidal thoughts/behaviors).

*Perceived mental health treatment need* was measured with the question, *“During the past 12* *months, was there any time you needed mental health treatment or counseling for yourself but didn’t get it?”* (yes = 1 and no = 0).

*Predisposing factors* were age group (21–25, 26–34, 35–49, 50–64, and 65+ years; the 35–49 age group was the reference category), gender, and race/ethnicity [Black, Hispanic, Asian, Other, and Non-Hispanic White (reference category)]. The NSDUH public use data sets do not provide chronological age to protect respondent anonymity.

*Enabling factors* were marital status (not married vs. married); educational level (less than high school, high school graduate, and more than high school [reference category]); income (<200% of the federal poverty line vs. ≥200% of the poverty line); employment status (not employed vs. employed); US military service that may allow access to Veterans’ Affairs/other military health insurance (ever served vs. did not serve); and health insurance gap (lacked insurance at some time in the past 12 months vs. always had insurance).

*Need factors* were major depressive episode (MDE) that met *DSM*-*IV* [27] criteria during the worst/most severe period of time, lasting 2 weeks or longer, in the past 12 months; and substance use disorder (SUD; alcohol and illicit drug abuse or dependence that met *DSM*-*IV* criteria) in the past 12 months. For sample description purposes only, we also examined substance abuse treatment use from any venue/modality (e.g., hospital, other inpatient/residential setting, emergency department, prison/jail, doctor’s/private therapist’s office, day treatment, self-help groups such as Alcoholics Anonymous or Narcotics Anonymous).

### Analysis

Analyses were conducted with Stata/MP 13′s svy function to account for NSDUH’s multi-stage, stratified sampling design. Stata’s subpop command was used for all subsample analyses to ensure that variance estimates incorporate the full sampling design. Following NSDUH guidelines, adjusted person-level analysis weights were created by dividing the final person-level analysis weights by the number of years of combined data (five in the current study). All estimates presented are weighted except for sample sizes. Chi-square tests were used to describe and compare sample characteristics of nonideators versus ideators and among the four groups of ideators. Binary logistic regression models were used to test H1a and H1b that groups that had planned and/or attempted suicide would have higher mental health treatment use and higher perceived treatment need, respectively, than the no plan/no attempt group with the four ideator groups as the independent variables and predisposing, enabling, and other need factors as covariates. H2, association of treatment use with predisposing, enabling, and need factors within each of the four ideator groups, was also tested with binary regression models. For the no plan/attempted group (n = 245), the number of covariates exceeded the recommended guidelines for the number of respondents exhibiting the less frequent outcome to degrees of freedom ratio (10:1) [28], which can potentially result in model overfitting but can be ameliorated by purposefully reducing the number of covariates [29]. Thus, the following adjustments were made: (1) age group was excluded (this variable was not significant in the full model); (2) race/ethnicity was dichotomized (all racial/ethnic minorities vs. non-Hispanic White); (3) alcohol and illicit drug use disorders were combined into one SUD variable.

## Results

### Sample characteristics of ideators versus nonideators

Table 1 shows that 3.5% of the sample reported past-year serious suicidal thoughts, and these ideators were more likely than nonideators to be aged 21–25 but less likely to be aged 65+ and more likely to be non-Hispanic White and of lower socioeconomic status (SES). Five percent of nonideators and 47% of ideators had MDE, and 8% of nonideators and 26% of ideators had SUD. Of those with any SUD, 9% of nonideators and 22% of ideators received substance abuse treatment in the preceding year. Eighteen percent of nonideators and 56% of ideators received mental health treatment, and 4% of nonideators and 35% of ideators perceived treatment need.

**Table 1:** Sample characteristics: nonideators versus ideators (%)

N (%)	Nonideators	Ideators	P
	147,575 (96.50)	7,348 (3.50)	
Age group			<.001
21–25 years	9.25	15.75	
26–34 years	16.70	19.21	
35–49 years	28.80	33.38	
50–64 years	26.78	23.05	
65+ years	18.47	8.61	
Male	48.07	46.69	.151
Race/ethnicity			<.001
Non-Hispanic White	68.04	73.55	
Black	11.38	10.52	
Hispanic	13.90	9.46	
Asian	4.70	3.38	
Other	1.98	3.08	
Not married	42.66	60.99	<.001
Education			<.001
<High school (HS)	14.25	14.62	
HS graduate	29.64	31.0	
Some college	25.25	29.33	
College graduate	30.86	25.05	
Not employed	34.35	42.67	<.001
Income <200% FPL^a^	31.55	43.46	<.001
Military service	11.38	11.17	.776
Health insurance coverage gap	5.04	9.31	<.001
Major depressive episode	5.10	46.97	<.001
Substance use disorders (SUD)	7.75	25.71	<.001
Alcohol abuse/dependence	6.44	20.41	<.001
Illicit drug abuse/dependence	1.85	10.71	<.001
Any substance abuse treatment use	1.39	7.78	<.001
Among those with SUD	9.23	21.46	<.001
Professional MH^b^ treatment use	12.61	50.01	<.001
Inpatient	0.52	8.17	<.001
Outpatient	5.82	32.36	<.001
Pharmacotherapy	10.65	44.57	<.001
Alternative MH treatment use	8.32	22.49	<.001
Any MH (professional and/or alternative) treatment use	17.89	55.93	<.001
Perceived MH treatment need	3.61	35.13	<.001
Without any treatment	2.10	24.49	<.001
With any treatment	14.09	45.76	<.001

^a^Federal poverty line.

^b^Mental health.

### Sample characteristics of four plan/attempt groups

Table 2 shows that of ideators, 69.8% neither made plans nor attempted suicide; 18.3% made plans but did not attempt suicide; 2.4% had no plan but attempted suicide; and 9.5% made plans and attempted suicide. The data further show that 27.8% of ideators made suicide plans and 11.9% of ideators attempted suicide. Of those who made suicide plans, 34.2% attempted suicide.

**Table 2:** Sample characteristics among four ideator groups (%)

N (%)	No plan/no attempt	Planned/no attempt	No plan/attempted	Planned/attempted	P
	4,985 (69.79)	1,331 (18.31)	245 (2.38)	787 (9.52)	
Age group					.075
21–25 years	15.55	13.45	24.42	19.54	
26–34 years	19.59	17.47	25.48	18.18	
35–49 years	32.85	38.12	31.28	28.64	
50–64 years	22.77	24.14	13.44	25.94	
65+ years	9.31	6.82	5.38	7.70	
Male	46.19	50.34	42.19	44.47	.166
Race/ethnicity					.010
Non-Hispanic White	74.50	77.06	58.60	63.57	
Black	10.14	8.60	16.17	15.57	
Hispanic	9.31	7.97	14.68	12.12	
Asian	3.13	3.16	7.03	4.79	
Other	2.92	3.21	3.52	3.95	
Not married	59.30	63.06	70.24	67.13	.020
Education					<.001
<High school (HS)	13.15	14.97	35.76	19.39	
HS graduate	28.93	30.92	21.66	31.14	
College graduate	26.60	24.97	10.23	17.55	
Not employed	38.43	52.89	51.98	51.87	<.001
Income <200% FPL^a^	40.32	46.10	63.13	56.44	<.001
Military service	10.8	12.78	10.49	10.93	.642
Health insurance coverage gap	9.11	9.06	12.74	9.38	.754
Major depressive episode	42.45	61.22	54.35	50.87	<.001
Substance use disorders (SUD)	24.44	26.58	38.44	30.17	.010
Alcohol abuse/dependence	19.54	20.17	26.11	25.77	.057
Illicit drug abuse/dependence	9.08	13.77	23.86	13.52	<.001
Any substance abuse treatment use	6.13	9.83	22.41	12.45	<.001
Among those with SUD	17.25	26.63	40.71	31.57	<.001
Professional MH^b^ treatment use	45.03	62.18	66.12	59.08	<.001
Inpatient	3.45	19.71	25.90	33.40	<.001
Outpatient	27.67	42.63	40.54	44.93	<.001
Pharmacotherapy	40.05	55.88	53.74	53.63	<.001
Alternative MH treatment use	21.0	26.21	20.34	26.82	.022
Any MH (professional and/or alternative) treatment use	51.67	66.74	69.45	63.0	<.001
Perceived MH treatment need	32.19	44.67	39.99	37.09	<.001
Without any treatment	23.45	35.17	26.92	17.23	<.001
With any treatment	43.10	50.44	46.69	50.83	.080

^a^Federal poverty line.

^b^Mental health.

The four ideator groups did not differ by age group, gender, military service, or health insurance coverage gap. However, the attempters (i.e., no plan/attempted group and planned/attempted group) had higher proportions of racial/ethnic minorities and those with lower SES than the non-attempters (i.e., no plan/no attempt group and planned/no attempt group). The no plan/no attempt group had the lowest rate (43%) of MDE, and the planned/no attempt group had the highest rate (61%). The four groups did not differ in alcohol use disorder, but the no plan/attempted group had the highest drug use disorder rate (24%).

The no plan/no attempt group also had a lower mental health treatment use rate (52%) than the other groups—67% for the planned/no attempt group, 70% for the no plan/attempted group, and 63% for the planned/attempted group. As expected, the attempters had higher rates of inpatient treatment use (26% for no plan/attempted group and 33% for planned/attempted group). The planned/no attempt group had a higher rate of perceived treatment need (45%) than the other groups; the no plan/no attempt group had a lower rate (32%). However, about half of those in all four groups who received mental health treatment still perceived treatment need, while only 17% (the planned/attempted group) to 35% (the planned/no attempt group) of those who did not receive treatment perceived need.

### Between-group differences in mental health treatment use and perceived need among ideators

Table 3 shows that relative to the no plan/no attempt group, the planned/no attempt, no plan/attempted, and planned/attempted groups had 1.5 [95% confidence interval (CI) 1.2–2.9], 2.5 (95% CI 1.4–4.3), and 1.6 (95% CI 1.3–2.2) times higher treatment use odds, respectively. Compared to the 35–49 age group, the 21–25 age group, 26–35 age group, and the 65+ age group had decreased treatment use odds. Being male, Black, Hispanic, Asian, not married, and having less education also decreased the odds, while having MDE increased treatment use odds by more than three times and a drug use disorder diagnosis increased the odds by 1.3. These findings support H1a.

**Table 3:** Between-group difference in mental health treatment use and perceived treatment need among ideators: odds ratios (OR) from logistic regression models

N	Treatment use vs. nonuse 7,348		Perceived treatment need vs. no perceived need 7,348	
	OR	95% CI^a^	OR	95% CI^a^
Planned/no attempt	1.49**	1.17–2.89	1.35*	1.06–1.73
No plan/attempted	2.45**	1.39–4.31	0.96	0.61–1.51
Planned/attempted	1.64**	1.25–2.15	1.03	0.75–1.42
No plan/no attempt	1.00		1.00	
Used mental health treatment (Did not use)			2.00***	1.67–2.39
Age 21–25 years	0.48***	0.39–0.58	1.07	0.88–1.31
Age 26–34 years	0.70**	0.55–0.90	1.32*	1.04–1.69
Age 50–64 years	0.96	0.75–1.23	0.55***	0.42–0.73
Age 65+ years	041***	0.29–0.59	0.27**	0.14–0.55
Age 35–49 years	1.00		1.00	
Male	0.49***	0.41–0.58	0.64***	0.52–0.78
Female	1.00		1.00	
Black	0.54***	0.43–0.69	0.92	0.72–1.18
Hispanic	0.55***	0.43–0.72	0.77	0.59–1.00
Asian	0.51*	0.28–0.92	0.45**	0.25–0.80
Other	0.81	0.56–1.17	0.87	0.60–1.25
Non-Hispanic White	1.00		1.00	
Not married	0.94	0.77–1.16	1.02	0.85–1.23
Married	1.00		1.00	
<High school	0.60***	0.47–0.77	0.94	0.70–1.26
High school graduate	0.73**	0.59–0.89	0.94	0.76–1.16
More than high school	1.00		1.00	
Not employed	1.71***	1.40–2.10	1.07	0.88–1.31
Employed	1.00		1.00	
Income <200% FPL^b^	0.92	0.77–1.08	1.11	0.93–1.34
(Income ≥200% FPL)	1.00		1.00	
Military service	1.27	0.90–1.78	1.22	0.86–1.73
No military service	1.00		1.00	
Health insurance coverage gap	1.08	0.83–1.40	1.34*	1.01–1.78
No gap	1.00		1.00	
Major depressive episode (MDE)	3.33***	2.79–3.96	2.25***	1.87–2.72
No MDE	1.00		1.00	
Alcohol use disorder	1.19	0.98–1.44	1.38**	1.10–1.72
No disorder	1.00		1.00	
Illicit drug use disorder	1.32**	1.08–1.60	1.66***	1.32–2.09
No disorder	1.00		1.00	

F(22,39) = 21.26, df = 60, P < .001 for treatment use; F(23,38) = 16.17, df = 60, P < .001 for perceived need.

* P < .05; ** P < .01; *** P < .001.

^a^Confidence interval.

^b^Federal poverty line.

Table 3 also shows that compared to the no plan/no attempt group, only the planned/no attempt group had higher odds of reporting perceived treatment need [1.4 (95% CI 1.1–1.7)]. Increased odds of perceived need were associated with mental health treatment use, the 26–34 age group, health insurance coverage gap, MDE, and alcohol and drug use disorders, while the 50–64 age group, the 65+ age group, males, and Asians had decreased odds. These finding partially support H1b.

### Factors associated with mental health treatment use within each ideator group

Table 4 shows that the 21–25 age group had lower treatment use odds in all but the no plan/attempted group. Males had lower odds in the no plan/no attempt and planned/no attempt groups but higher odds in the no plan/attempted group. In the no plan/attempted group, all minorities combined had lower odds; in the other three groups, in which analysis by each major ethnic group was possible, Blacks and Hispanics, but not Asians or others, had lower odds. Those with less education and nonemployed status also had lower odds in the no plan/no attempt and planned/no attempt groups; lower income also decreased the odds in the no plan/no attempt group, while military service increased the odds in this group. MDE significantly increased the odds in all but the no plan/attempted group. SUDs were not significant predictors in any group. These findings partially support H2.

**Table 4:** Factors associated with any mental health treatment use vs. nonuse within each plan/attempt group: logistic regression models

N	No plan/no attempt 4,985		Planned/no attempt 1,331		No plan/attempted 245		Planned/attempted 787	
	OR	95% CI^a^	OR	95% CI^a^	OR	95% CI^a^	OR	95% CI^a^
Age 21–25 years	0.48***	0.37–0.62	0.48**	0.28–0.80			0.38**	0.21–0.69
Age 26–34 years	0.66*	0.48–0.92	0.78	0.47–1.32			0.87	0.40–1.92
Age 50–64 years	0.84	0.63–1.12	1.45	0.66–3.16			1.38	0.49–3.95
Age 65+ years	0.37***	0.24–0.58	0.77	0.26–2.25			0.60	0.20–1.82
Age 35–49 years	1.00		1.00				1.00	
Male	0.45***	0.37–0.54	0.44***	0.29–0.66	3.53**	1.42–8.77	0.66	0.38–1.15
Female	1.00		1.00		1.00		1.00	
Black	0.69*	0.52–0.91	0.31*	0.13–0.76			0.28*	0.10–0.73
Hispanic	0.61**	0.45–0.83	0.53*	0.31–0.91			0.21**	0.08–0.54
Asian	0.57	0.28–1.14	0.66	0.20–2.12			0.19	0.03–1.04
Other	0.78	0.48–1.27	0.91	0.38–2.15			0.43	0.13–1.43
Non-Hispanic White	1.00		1.00				1.00	
All other race/ethnic groups					0.38*	0.17–0.89		
Non-Hispanic White					1.00			
Not married	0.86	0.67–1.09	1.27	0.77–2.09	0.62	0.24–1.63	1.11	0.52–2.38
Married	1.00		1.00		1.00		1.00	
<High school	0.63**	0.47–0.85	0.47*	0.23–0.96	0.37	0.11–1.32	0.64	0.27–1.54
High school graduate	0.71**	0.55–0.91	0.87	0.58–1.31	0.34	0.10–1.13	0.68	0.31–1.49
>High school	1.00		1.00		1.00		1.00	
Not employed	1.65***	1.29–2.10	2.06**	1.38–3.06	1.66	0.63–4.36	2.02	0.95–4.28
Employed	1.00		1.00		1.00		1.00	
Income <200% FPL^b^	0.82*	0.67–0.99	1.04	0.69–1.59	0.71	0.24–2.12	1.60	0.83–3.10
Income ≥200% FPL	1.00		1.00		1.00		1.00	
Military service	1.48*	1.01–2.19	0.83	0.36–1.92	1.06	0.19–6.04	0.38	0.10–1.43
No military service	1.00		1.00		1.00		1.00	
Health insurance coverage gap	1.06	0.77–1.45	0.82	0.47–1.45	0.81	0.14–4.71	2.32**	1.31–4.12
No gap	1.00		1.00		1.00		1.00	
MDE	3.20***	2.57–3.98	3.33***	2.37–4.68	1.87	0.73–4.68	8.15***	4.43–14.99
No MDE	1.00		1.00		1.00		1.00	
Alcohol use disorder	1.24	1.00–1.55	0.84	0.52–1.36			0.76	0.33–1.71
No disorder	1.00		1.00				1.00	
Illicit drug use disorder	1.19	0.92–1.53	1.63	0.93–2.87			2.34	0.79–6.91
No disorder	1.00		1.00				1.00	
Alcohol or drug use disorder					1.79	0.79–4.02		
No disorder					1.00			

F(19,42) = 20.89, df = 60, P < .001 for the no plan/no attempt group; F(19,42) = 6.76, df = 60, P < .001 for the planned/no attempt group.

F(11,49) = 2.37, df = 59, P = .019 for the no plan/attempted group; and F(19,42) = 6.55, df = 60, P < .001 for the planned/attempted group.

* P < .05; ** P < .01; *** P < .001.

^a^Confidence interval.

^b^Federal poverty line.

## Discussion

Building on previous research on mental health treatment use among individuals with suicidal ideation, this study examined factors associated with mental health treatment use and perceived treatment need among four groups of ideators of US adults aged 21+. The 3.5% prevalence rate of suicidal ideation in the present study appears to be comparable to the 3.3% prevalence rate (among those aged 18–54) found in the 2001–2003 NCS-R [6]. The 27.8% rate of suicide plans among ideators and the 34.2% attempt rate among planners in the present study are also comparable to the 28.6 and 32.8% rates, respectively, found in the NCS-R [6]. The lack of notable decreases in the plan and attempt rates over time is a significant concern. The 11.9% attempt rate among ideators in this study is especially striking, given that a nonfatal suicide attempt is the strongest known clinical predictor of eventual suicide [30, 31].

As expected, all four groups of ideators had high MDE and SUD rates relative to nonideators. The 38% SUD rate among the no plan/attempted group implicates substance misuse as a trigger or substance-induced impulse control problems as a contributor to suicidal behaviors [32]. However, more than half of those with SUD in the no plan/attempted group and significantly higher proportions in the other three groups had not received SUD treatment. Untreated SUDs are likely to pose a continued risk for those with serious suicidal ideation.

About 50 to 70% of the ideators used mental health treatment, and about half of all treatment users perceived a need for (more) treatment, indicating that the treatment received was inadequate for the severity of symptoms among those at risk of suicide and that treatment effectiveness needs to be improved for those receiving treatment. The high rate of perceived need among those who received treatment may also stem from heightened motivation from treatment. The finding that only a small proportion of those who received no treatment perceived a need for it even given their suicide attempt further supports the need to increase outreach to untreated individuals with suicidal ideation to prevent attempts. Although mental health service use rates have steadily increased in the general population aged 18+ from 13.0% in 2002 to 14.5% in 2012 [33], the slow and incremental increase appears to have left out many people who were in great need of treatment. Given that as many as 46% of individuals aged 18+ years who perceived a need for treatment but did not get it reported treatment cost as a barrier [33], treatment should also be made affordable.

Confirming previous research and in line with Andersen’s behavioral model of health service use, predisposing and enabling factors were also significantly associated with mental health service use. The lower rates of treatment use and perceived treatment need among younger and older individuals, men, racial/ethnic minorities, and low-SES individuals at risk of suicide reflect the lower rates of mental health service use among individuals sharing the same sociodemographic characteristics in the general population. For example, among US adults (aged 18+), 10.2% of Blacks and 7.1% of Hispanics, compared to 17.8% of non-Hispanic Whites, used any mental health services in 2012 [33]. As summarized [17–22], stigma about mental illnesses, self-sufficiency beliefs, religious coping, negative attitudes toward professional mental health treatment use, and affordability issues are likely barriers to accessing treatment among racial/ethnic minority groups. Continued efforts are needed to make mental health treatment accessible and affordable for all those at risk of suicide.

This study’s key findings are that the four ideator groups have different rates of treatment access and perceived treatment need that are not commensurate with their risk level. Specifically, the planned/attempted group, which appears to be at the highest suicide risk, was not the group that accessed treatment or perceived treatment need most. Only one-third of this group received inpatient treatment (some may have received it only after they attempted suicide), more than 40% did not access any professional mental health providers either before or after their suicide attempt, and more than 80% of those who did not access treatment did not perceive treatment need. Further research should examine if low treatment access rates and perceived need in this group stems from higher levels of hopelessness and determination to end their lives or from more systemic access barriers, especially among those with low SES. Treatment programs that specifically target high-risk groups in a timely manner (i.e., before an attempt) are needed.

Controlling for predisposing, enabling, and other need factors, the no plan/attempted group was most likely to use treatment (again, some may have received it only after they attempted suicide), but one-third of this group did not access any professional mental health treatment. The rate of perceived need among these nonusers was also disconcertingly low. The planned/no attempt group included the largest proportion of people who perceived treatment need, and the no plan/no attempt group (the largest group of ideators) perceived treatment need as much as the other groups. Data constraints precluded examination of whether or not treatment prevented some individuals in the planned/no attempt group from carrying out their suicide plans and helped those in the no plan/no attempt group make no plans/attempts. Large audits of completed suicides in England and Wales and in Australia found that 20% of suicides could have been prevented with effective treatment [34, 35]. We need to continue to examine the potential benefits of treatment in preventing suicidal acts among suicide ideators and planners and improve treatment effectiveness.

The study had the following limitations stemming from data constraints: (1) All suicide-related data were self-reports and not based on validated screening/assessment tools; thus stigma may have resulted in underreporting; (2) the data set did not include the following variables: frequency of serious suicidal thoughts, plans, and attempts; the severity of the thoughts/behaviors; known risk or protective factors for suicidal thoughts/behaviors such as perceived social support, hopelessness, previous suicide attempts, and diagnosis of psychiatric disorders other than MDE and SUD; and attitudes and beliefs associated with mental health treatment use; (3) the use of cross-sectional data prohibited establishing the temporal order between suicidal thoughts/behavior and treatment; and (4) treatment adequacy/effectiveness could not be examined.

## Conclusions

Despite its limitations, the study contributes to expanding the knowledge base about suicidal ideation and behaviors and suggests the following for clinical assessment and mental health and SUD treatment access: (1) High MDE and SUD rates among ideators underscore the importance of suicide risk assessment for those with mental health problems and/or SUDs in both primary care and specialty settings. (2) Given that 30% of those with serious suicidal thoughts made suicide plans and/or attempted suicide but that more than 30% of them received no mental health treatment either before or after planning or attempting also underscores the importance of mental health treatment access for those at-risk of suicide, especially racial/ethnic minorities and those of low SES. (3) The finding that almost 12% of those with serious suicidal thoughts made nonfatal suicide attempts and that racial/ethnic minorities were overrepresented among the attempters highlights the importance of prevention strategies targeting these high-risk groups. Community-based suicide-prevention education and outreach efforts to motivate them to seek help are important steps that must be taken in tandem with accessible and affordable mental health and SUD treatment services.
